# Effects of Cadmium Exposure on Leydig Cells and Blood Vessels in Mouse Testis

**DOI:** 10.3390/ijerph19042416

**Published:** 2022-02-19

**Authors:** Shi-Han Yang, Si-Ting Chen, Chen Liang, Yong-Hong Shi, Qiu-Sheng Chen

**Affiliations:** 1 College of Resource and Environment Science, Nanjing Agricultural University, Nanjing 210095, China; yangshihan2020@126.com; 2 College of Veterinary Medicine, Nanjing Agricultural University, Nanjing 210095, China; qiushengliuyi@sohu.com; 3 College of Veterinary Medicine, South China Agricultural University, Guangzhou 510642, China; 15702094622@163.com (S.-T.C.); liangchen52@126.com (C.L.)

**Keywords:** cadmium, testis, blood vessel, endothelium

## Abstract

Environmental exposure to cadmium (Cd) contributes to a decline in the quality of human semen. Although the testis is sensitive to Cd exposure, the mechanism underlying how cadmium affects the testis remains to be defined. In this study, male mice were treated with intraperitoneal injections of 0, 0.5, 1.5 and 2.5 mg CdCl_2_/kg/day for 10 days, respectively. Both the testicular weight and the 3β-HSD activity of Leydig cells were significantly reduced with the administration of 2.5 mg CdCl_2_/kg/day. The height of endothelial cells in the interstitial blood vessels significantly increased with the use of 2.5 mg CdCl_2_/kg/day compared with the control. Western blot data showed that the protein levels of CD31, αSMA, caveolin and Ng2 increased with cadmium exposure, and this increase was particularly significant with the administration of 2.5 mg CdCl_2_/kg/day. CD31, αSMA, caveolin and Ng2 are related to angiogenesis. Based on our data, cadmium exposure may stimulate the proliferation of the mural cells and endothelial cells of blood vessels, which may lead to abnormal function of the testis.

## 1. Introduction

Reproduction is an important process for the survival and development of a species. Recent data indicate that infertility occurs in 15% of couples who are of reproductive age, including ovulatory dysfunction, male factor infertility, and tubal disease [1,2]. Subfertility and sterility are male reproductive disorders, which are related to the environment [3]. Male causes of infertility are responsible for 40–50% of all cases of infertility [4]. Infertile men usually have impaired spermatogenesis, such as asthenospermia and oligozoospermia [5]. The causes of male infertility are complex and remain largely unknown. There has been a significant decline in male fertility and semen quality in recent decades. Exposure to environmental and occupational pollution has been linked to the quality of male semen [6]. Environmental exposure to cadmium (Cd) contributes to a decline in the quality of human semen [7].

Cadmium is a by-product and environmental contaminant from industrial processes and agricultural activities [1]. Cadmium is a heavy metal and a major environmental toxicant. Human beings are mainly exposed to Cd from drinking water and food [8]. Occupational exposure usually occurs in industries involving mining, batteries, pigments, coatings, electroplating and plastic stabilizers. Sources of cadmium exposure include plastic toys, battery, paints, ceramics, contaminated water, air, soil, food, fertilizers, and cigarette smoke [9,10]. 

Cadmium can cause serious damage to human health, and is believed to be a human carcinogen [11]. Cadmium has a long biological half-life (10–35 years) and a low rate of excretion, and, therefore, it easily accumulates in different human organs [3,12]. Although cadmium is a strong toxicant, it possesses no biological functions in human beings [12].

The testis contains two compartments (seminiferous tubules and intertubular tissue) [13]. In seminiferous tubules, germ cells in various stages of development (spermatogonia, spermatocytes, spermatids, spermatozoa) develop under the regulation of the somatic Sertoli cells [14]. Leydig cells are the major steroidogenic cell population of the testicular interstitium and function primarily to produce androgens [15]. In rat testis, Cd treatment leads to decrease of serum testosterone concentration and change of sperm parameters [16]. Cd exposure has been reported to be associated with vascular disorders. Plasma malondialdehyde and protein carbonyl groups increased while the erythrocytic glutathione decreased in Cd-exposed subjects [17]. Cadmium exposure causes hypertension and vascular damage through activating NADPH oxidase, renin-angiotensin system, and cyclooxygenase 2 pathways in arteries [18].

Recent studies have shown that the testis is extremely sensitive to cadmium toxicity, due to the high expression of ZIP8 in the endothelial cells of the testis. ZIP8 is mainly involved in the transport of zinc, manganese, and cadmium from the extracellular space [12,19]. Although cadmium can cause various forms of damage to the testis [3,8,20], recent data have shown that it primarily targets the vascular endothelium in the testis [21]. However, the mechanism underlying how cadmium affects the vascular system of the testis remains to be defined. The aim of this study was to examine the effect of cadmium exposure on mouse testis through multiple approaches. In this study, male mice were administered different doses of cadmium daily for 10 days. The blood- vessel-associated proteins significantly increased with a high dose of cadmium, suggesting that the proliferation of mural cells or endothelial cells was stimulated.

## 2. Material and Methods

### 2.1. Animals and Tissue Collection

All animal experiments were approved by the Institutional Animal Care and Use Committee of South China Agricultural University (#2019-0136). Adult male CD1 mice (5 weeks old, body weight 30 ± 1 g) were maintained in a temperature- and light-controlled specific pathogen-free fertility room (12 h light:12 h dark cycle) and randomly divided into 4 groups, with 8 mice per group. 

Based on previous studies [22,23,24], male mice were treated with 0, 0.5, 1.5 mg/kg and 2.5 mg/kg CdCl_2_. CdCl_2_ (#202908, Sigma, St. Louis, MO, USA) was dissolved in 0.85% NaCl saline and injected i.p. in a volume of 100 μL daily for 10 days. Ten days after cadmium exposure, testes were removed from treated mice, weighed, and fixed in neutral buffered formalin and embedded in paraffin.

### 2.2. Immunohistochemistry

Histochemistry was performed as previously described [25]. Testes were fixed in neutral buffered formalin and embedded in paraffin. Paraffin sections (5 μm) were deparaffinized and hydrated. Antigen retrieval was achieved by microwaving the sections in 10 mM sodium citrate buffer (pH 6.0). After the activity of endogenous horseradish peroxidase was inhibited with 3% H_2_O_2_ for 15 min, sections were blocked with 10% normal horse serum in PBS. Following this, sections were incubated with rabbit anti-Ng2 (1:200, ab275024, Abcam, Waltham, MA, USA), rabbit anti-caveolin-1 (1:200, sc-894, Santa Cruz, Dallas, TX, USA), rabbit anti-Cd31 (1:200, ab28364, Abcam) or rabbit anti-αSMA (1:200, 19245T, Cell Signaling Technology, Danvers, MA) overnight at 4 °C. After sections were incubated with a biotin-labeled goat anti-rabbit IgG antibody and a streptavidin-conjugated HRP complex (Zhongshan Golden Bridge, Beijing, China), the signals were visualized with the DAB Horseradish Peroxidase Color Development Kit. The sections were counterstained with hematoxylin. Images were obtained by microscope (Leica, DM2500, Wetzlar, Germany) with LAS V4.3 system. 

### 2.3. Western Blot

Western blot was carried out as previously described [26]. Briefly, testis tissues were frozen and lysed in lysis buffer (50 mM Tris-HCl, pH 7.5; 150 mM NaCl; 1% Triton X-100; 0.25% sodium deoxycholate). Protein samples were quantified with a bicinchoninic acid assay reagent kit(Thermo Fisher Scientific, Waltham, MA, USA), separated by SDS polyacrylamide gel electrophoresis and transferred onto a PVDF membrane (Merck KGaA, Darmstadt, Germany). Membranes were blocked in 5% nonfat dry milk and incubated overnight at 4 °C with rabbit anti-Ng2 (1:1000, ab275024, Abcam), rabbit anti-caveolin-1 (1:1000, sc-894, Santa Cruz), rabbit anti-CD31 (1:1000, ab28364, Abcam, Waltham, MA, USA), rabbit anti-αSMA (1:1000, 1:200, 19245T, Cell Signaling Technology, Danvers, MA) or rabbit anti-tubulin (1:1000, 2144S, Cell Signaling Technology), respectively. After the membrane was incubated with HRP-conjugated secondary antibody, signals were detected by ECL chemiluminescent kit (Merck Millipore, Billerica, MA). Five male mice were used in each group for Western blot analysis.

### 2.4. Transmission Electron Microscopy (TEM)

The preparation of testis for TEM observation was performed as previously described [27]. Briefly, testis was cut into small parts, fixed in 2.5% glutaraldehyde and 1% osmium tetroxide, and embedded in Araldite, respectively. Ultrathin sections (50 nm) were stained with 1% uranyl acetate and Reynold’s lead citrate for transmission electron microscopy examination (TEM; Hitachi H-7650, Tokyo, Japan).

### 2.5. Enzyme Histochemistry for 3beta-Hydroxysteroid Dehydrogenase (3beta-HSD)

The activity of 3β-HSD was shown as previously described [28]. Testicular tissues were frozen in liquid nitrogen and stored at –80 °C. Frozen sections (10 μm) were then cut and heat fixed at 50 °C for 2 min. Following 3 washes in PBS (0.1 M, pH 7.6), sections were incubated with substrate mixture containing 11.9 mL PBS, 900 μL nitroblue tetrazolium (NBT; 1.0 mg NBT dissolved in 1 mL distilled water), 600 μL DHEA (4 mg dissolved in 1 mL dimethyl sulfoxide) and 20.0 mg NAD in dark at 37 °C. Sections were washed in PBS, fixed with 10% neutral buffered formalin and mounted in gelatin. Images were obtained by microscope (Leica, DM2500, Wetzlar, Germany) with LAS V4.3 system.

### 2.6. Statistical Analysis 

Data are shown as mean ± s.e.m. Statistical analysis was performed with an unpaired Student’s *t*-test or two-way ANOVA followed with Bonferroni post hoc test. At least three independent repeats were performed in all groups. *p* < 0.05 was considered statistically significant.

## 3. Results

After the male mice were treated with different doses of cadmium, it was observed that the weights of the testes were slightly decreased in the 1.5 mg/kg group and significantly decreased in the 2.5 mg/kg group, from 33.41 ± 1.32 to 31.19 ± 1.54 mg. In the 2.5 mg/kg group, the testes became congestive and exhibited a light red color. No change was detected in the 0.5 mg/kg group compared with the control.

### 3.1. Ultrastructural Changes

**Figure 1:**
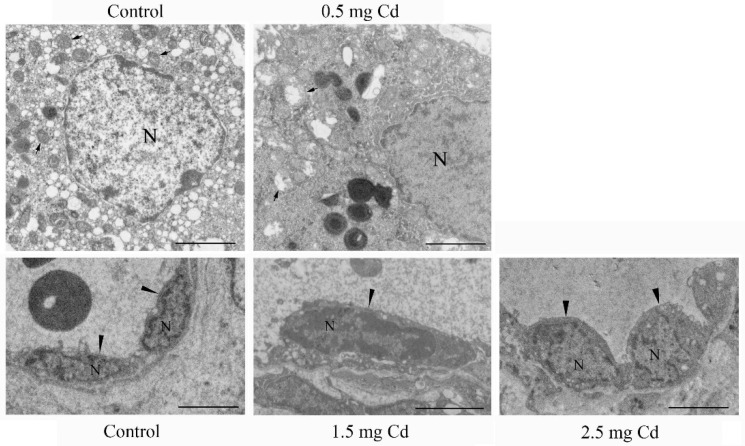
The ultrastructure of mouse testes treated with 0.5 mg/kg and 2.5 mg/kg CdCl_2_ for 10 days. Three mice were used in each group for analysis. N, nucleus; arrow, mitochondium; arrowhead, endothelial cells. Bar, 2 μm.

Under TEM, the mitochondria of the Leydig cells became disorganized, and vesicles were pronounced in the 0.5, 1.5 and 2.5 mg/kg groups compared with the control. In the control group, vascular endothelial cells were flat and attached to the vessel wall. However, the height of the endothelial cells significantly increased in the 1.5 mg/kg and 2.5 mg/kg groups (Figure 1). 

### 3.2. Enzymic Activity of 3β-HSD

**Figure 2:**
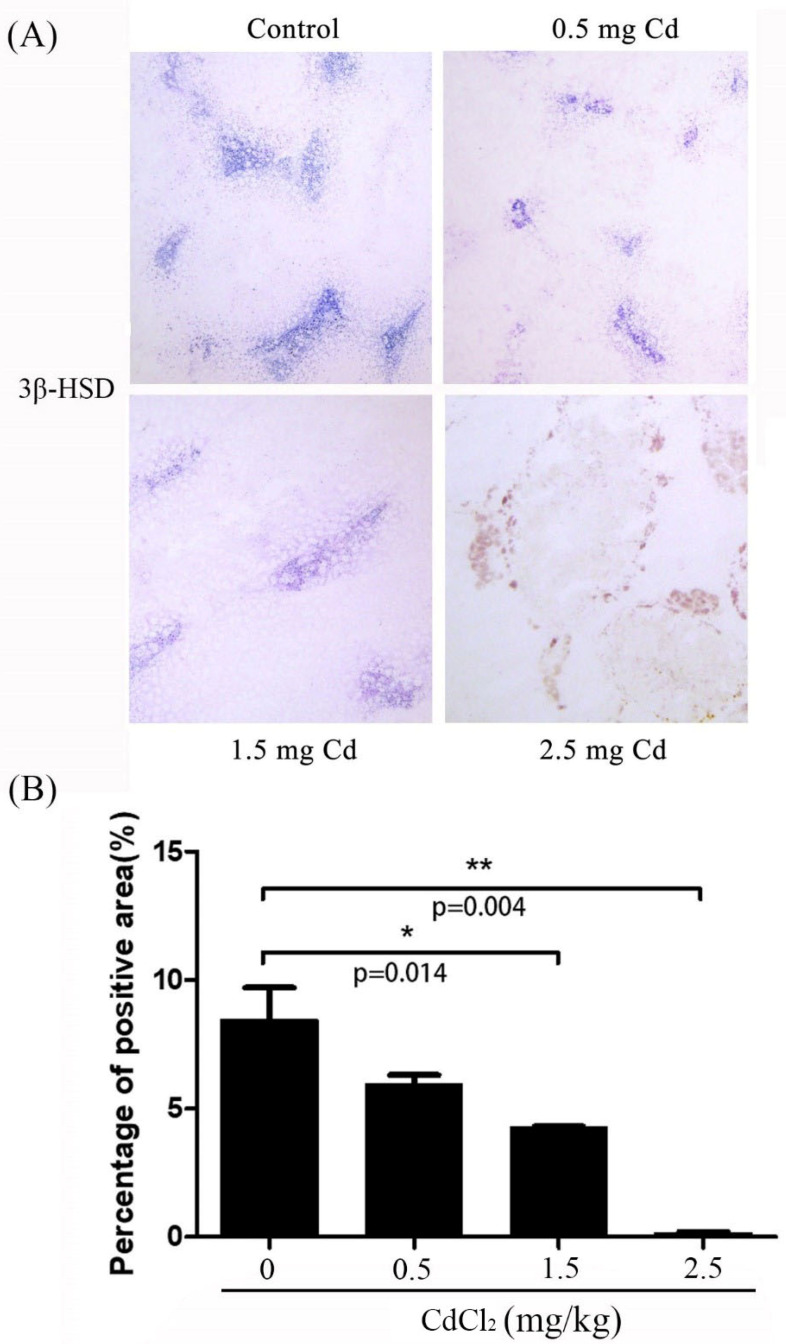
The histochemical staining of 3β-HSD in mouse testes treated with 0, 0.5, 1.5 and 2.5 mg/kg CdCl_2_ daily for 10 days, respectively. (**A**) 3β-HSD histochemistry. (**B**) The semiquantitative density of 3β-HSD. Three mice were used in each group. Bar, 70 μm. * *p* < 0.05; ** *p* < 0.01.

Leydig cells are responsible for the production of the male sex hormone testosterone; 3β-HSD is a marker for the steroidogenesis of Leydig cells [29]. In the control group, 3β-HSD staining was strongly detected in the interstitial compartment of the testis (8.10 ± 0.78). The staining was slightly decreased in the 0.5 and 1.5 mg/kg groups (5.23 ± 0.63 and 3.16 ± 0.20), but disappeared in the 2.5 mg/kg group (0.23 ± 0.03) (Figure 2).

### 3.3. αSMA Immunostaining and Protein Level

**Figure 3:**
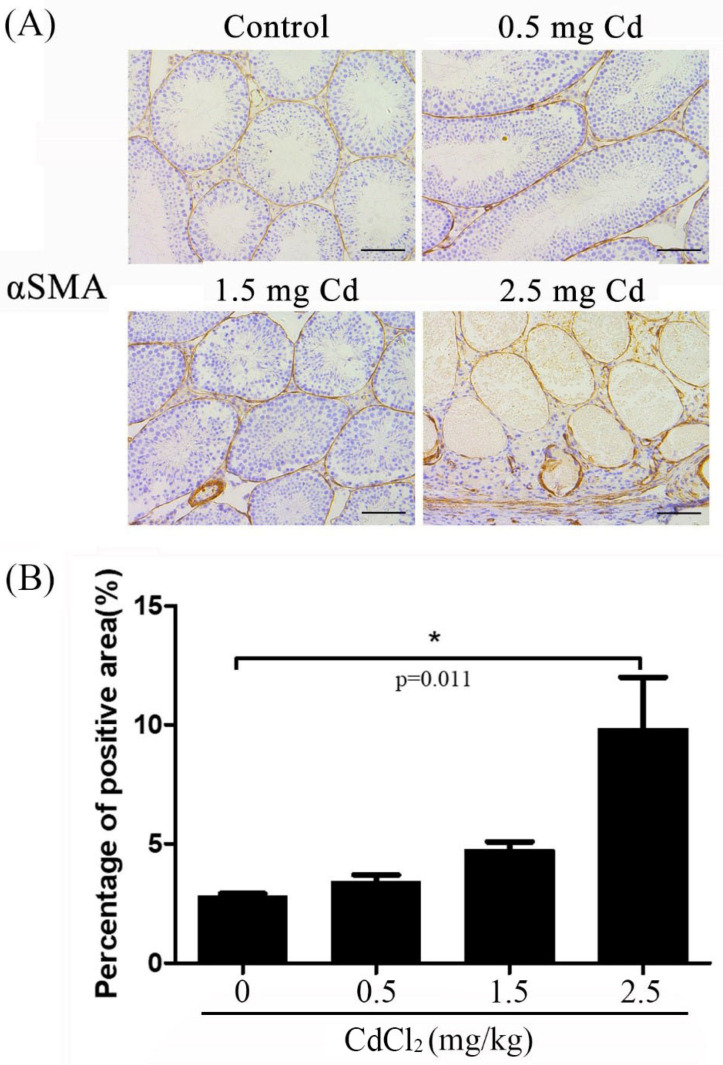
αSMA immunostaining in mouse testes treated with 0, 0.5, 1.5 and 2.5 mg/kg CdCl_2_ daily for 10 days, respectively. (**A**) αSMA immunostaining. (**B**) The semiquantitative density of αSMA immunostaining. Three mice were used in each group. Bar, 100 μm. * *p* < 0.05.

**Figure 4:**
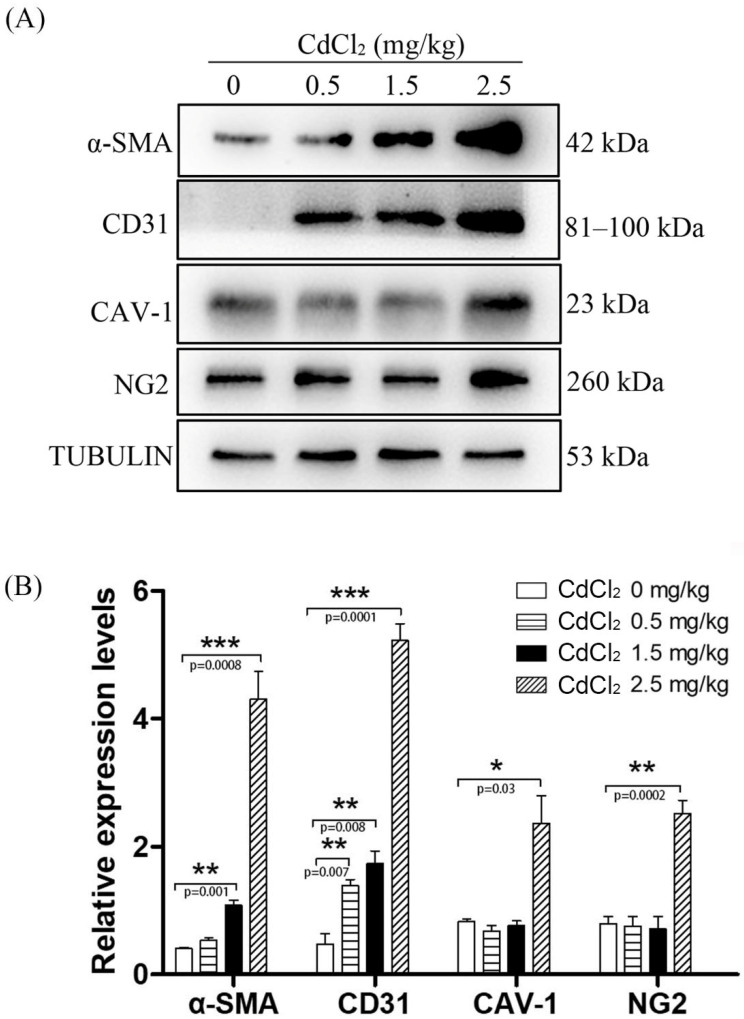
Western blot of αSMA, CD31, caveolin-1 and NG2 protein levels in mouse testes treated with 0, 0.5, 1.5 and 2.5 mg/kg CdCl_2_ daily for 10 days, respectively. (**A**) Western blot analysis. (**B**) The semiquantitative density of each protein band. Five mice were used in each group. * *p* < 0.05; ** *p* < 0.01; *** *p* < 0.001.

It has been demonstrated that αSMA is a marker for peritubular myoid cells and myocytes of the blood vessels in the testes [30]. Both peritubular myoid cells and myocytes of the blood vessels are similar to smooth muscle cells [31]. In the control group, weak αSMA immunostaining could be observed in the blood vessels and peritubular cells (2.73 ± 0.08), which gradually became stronger. Furthermore, strong αSMA staining could be observed in the peritubular myoid cells, myocytes, and smooth muscle cells of the testis in the 2.5 mg/kg group (9.16 ± 1.41) (Figure 3). Western blot also showed an increasing trend for αSMA protein levels when the dose of cadmium increased from 0.5 mg/kg to 2.5 mg/kg (Figure 4).

### 3.4. CD31 Immunostaining

**Figure 5:**
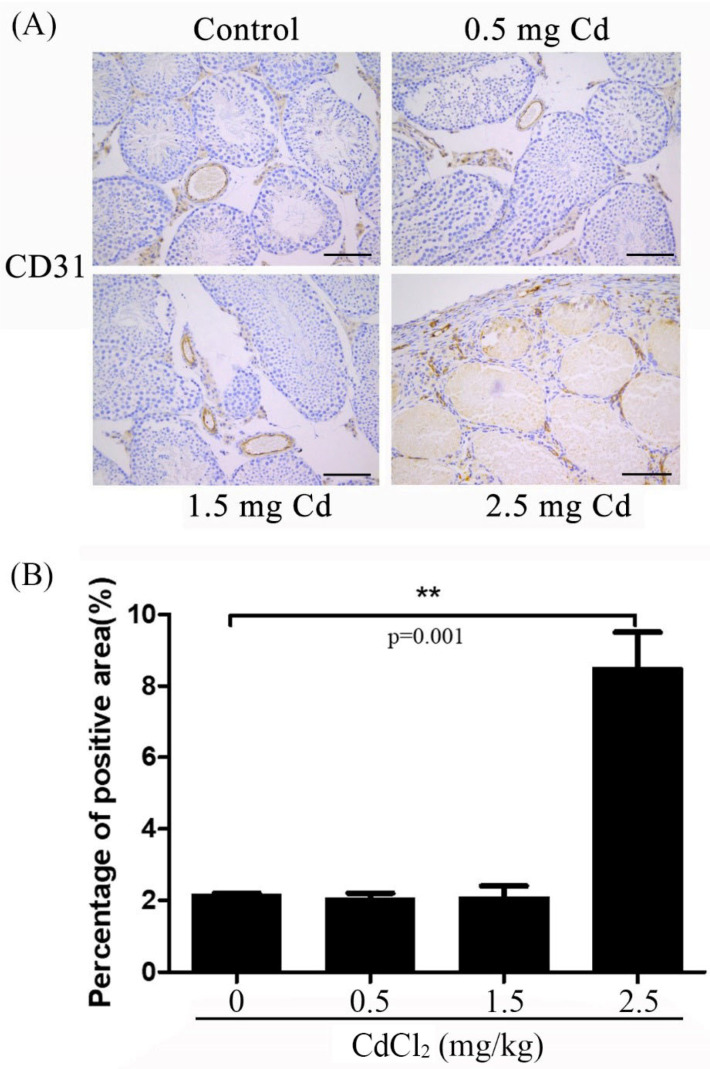
CD31 immunostaining in mouse testes treated with 0, 0.5, 1.5 and 2.5 mg/kg CdCl_2_ daily for 10 days, respectively. (**A**) CD31 immunostaining. (**B**) The semiquantitative density of CD31 immunostaining. Three mice were used in each group. Bar, 100 μm. ** *p* < 0.01.

CD31 is a reliable marker for vascular endothelial cells [32]. In comparison with the control (2.16 ± 0.03), the level of CD31 staining in the interstitial blood vessels was similar in both the 0.5 and 1.5 mg/kg groups (2.03 ± 0.08 and 2.10 ± 0.07). However, the level of CD31 staining significantly increased in the tubules and interstitial vessels (Figure 5). Western blot also showed that CD31 staining was significantly increased in the 0.5 and 1.5 mg/kg groups compared with the control. A strong level of CD31 staining was observed in the 2.5 mg/kg group (8.56 ± 0.58) (Figure 4).

### 3.5. Caveolin-1 Immunostaining

**Figure 6:**
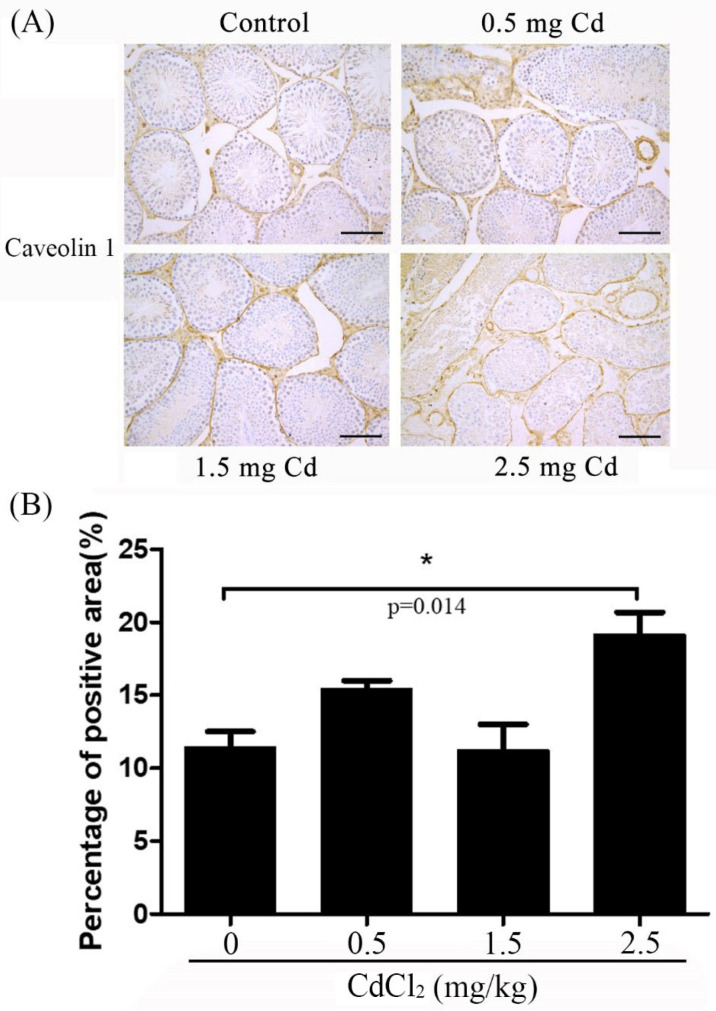
Caveolin-1 immunostaining in mouse testes treated with 0, 0.5, 1.5 and 2.5 mg/kg CdCl_2_ daily for 10 days, respectively. (**A**) Caveolin-1 immunostaining. (**B**) The semiquantitative density of caveolin-1 immunostaining. Three mice were used in each group. Bar, 100 μm. * *p* < 0.05.

Caveolin-1 is located in the blood vessels and is closely related to angiogenesis, potentially contributing to the progress of this process [33]. Cav-1 knockout mice show a significant increase in BRB permeability [34]. In the control group, caveolin-1 staining was weakly detected in the interstitial vessels (11.36 ± 0.61). Caveolin-1 staining signals became stronger in the interstitial vessels in both the 0.5 and 1.5 mg/kg groups (15.50 ± 0.32 and 11.16 ± 1.01). There was also a strong caveolin-1 staining signal detected in the peritubular cells and interstitial compartment (Figure 6). Immunostaining and Western blot showed that caveolin-1 levels were significantly increased in the 2.5 mg/kg group compared with the other groups (19.40 ± 0.92 for immunostaining) (Figure 4).

### 3.6. NG2 Immunostaining

**Figure 7:**
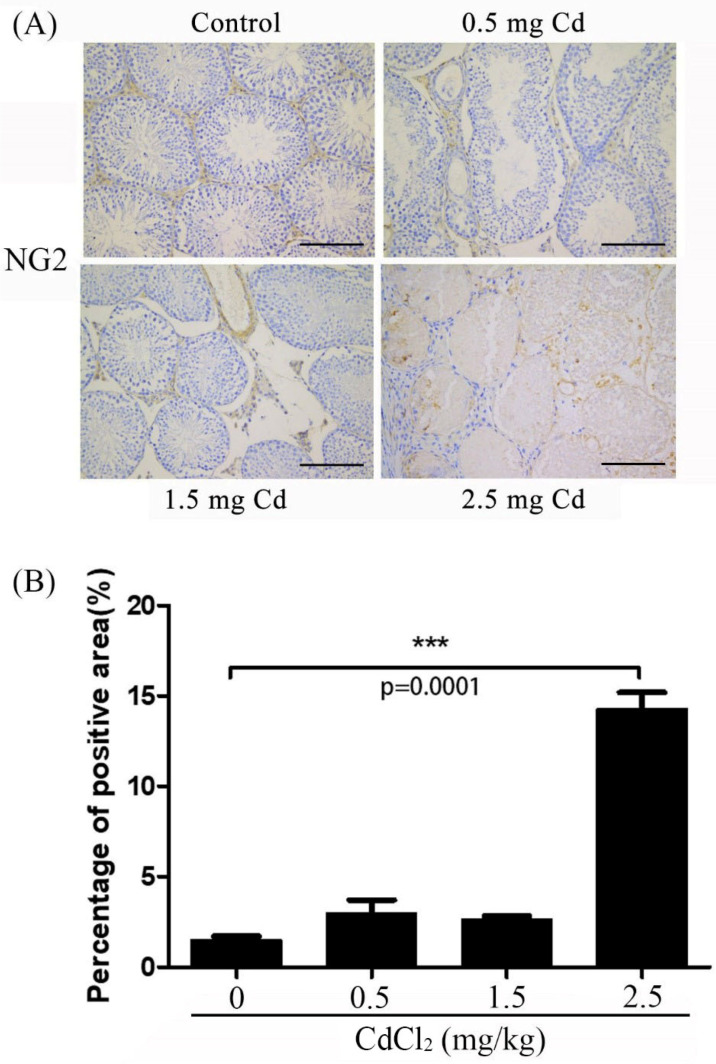
NG2 immunostaining in mouse testes treated with 0, 0.5, 1.5 and 2.5 mg/kg CdCl_2_ daily for 10 days, respectively. (**A**) NG2 immunostaining. (**B**) The semiquantitative density of NG2 immunostaining. Three mice were used in each group. Bar, 100 μm. *** *p* < 0.001.

NG2, a chondroitin sulfate proteoglycan, is a marker for the pericytes of blood vessels [30,35]. Compared with the control (1.50 ± 0.11), a similar weak NG2 staining signal was detected in the interstitial compartment in the 0.5 and 1.5 mg/kg groups (2.83 ± 0.44 and 2.66 ± 0.08). The NG2 staining signal became stronger in the testicular tubules and interstitial space (Figure 7). Immunostaining and Western blot also showed that NG2 levels were significantly increased in the 2.5 mg/kg group compared with the other groups (14.30 ± 0.51 for immunostaining) (Figure 4).

## 4. Discussion

In our study, the weight of the testes was significantly decreased in the 2.5 mg/kg group, but CdCl_2_ at doses of 0.5 and 1.5 mg/kg had no significant effects. Under EM observation, the morphology of mitochondria was disorganized, even in the 0.5 mg/kg CdCl_2_ group. Recent studies have indicated that the testes are extremely sensitive to Cd toxicity [12]. When adult male Sprague–Dawley rats were treated with 5, 10 and 15 mg CdCl_2_/kg/day for 17 days by oral gavage, their testes showed adverse structural effects to areas including the tunica albuginea, tubular lumen, and interstitial space among the seminiferous tubules [36]. Cadmium treatment also caused severe structural damage to the seminiferous tubules, Sertoli cells and blood–testis barrier [8].

The vasculature of the testes is important for their endocrine function [37]. Pericytes and vascular smooth muscle cells are responsible for maintaining the structural and functional stability of blood vessels [38]. Previous studies have shown that the testicular endothelium is sensitive to the damage caused by Cd treatment because Cd-induced ZIP8 expression in the endothelial cells is responsible for the accumulation of Cd [19,39]. Cd treatment causes the decrease of serum testosterone concentration and change of sperm parameters in rat testis [16]. In Cd-exposed population, plasma malondialdehyde and protein carbonyl groups increase while the erythrocytic glutathione decreases [17]. Cadmium exposure also causes hypertension and vascular damage [18].

In our study, we found a significant increase in vascular vessel-related molecules (CD31, NG2, αSMA and caveolin-1) after the mice were treated with a high dose of CdCl_2_/kg. In comparison with the control, the height of testicular endothelial cells was significantly increased and enlarged in the blood vessels. Even in the low-dosage groups, there was also a slight increase in the protein levels of CD31, NG2, αSMA and caveolin-1. The effects of Cd treatment on αSMA expression and the proliferation of myofibroblasts were also reported in the lung and kidney [40,41,42]. Elevated levels of α-SMA are observed in chronic obstructive pulmonary disease (COPD) patients with higher circulatory Cd [43]. Although there are no reports regarding the capillarization of Cd, arsenic treatment promotes the capillarization of the sinusoidal endothelium in the liver [39]. Both epidemiological studies and animal studies have shown that Cd exposure is related to atherosclerosis, hypertension, and endothelial damage, which are responsible for cardiovascular diseases [39,44]. Cd exposure induces hypertension and impairs vascular responses to phenylephrine, acetylcholine, and sodium nitroprusside [45]. It is possible that Cd-induced proliferation of vessel endothelial cells or mural cells may cause hypertension.

Leydig cells (LCs) are present in the interstitial compartment of the testis, and are responsible for the synthesis of androgens [46]. The steroidogenic enzyme 3beta-hydroxysteroid dehydrogenase (3β-HSD) is a marker for the steroidogenesis of Leydig cells [47]. The damage caused by cadmium to Leydig cells has been reported in several studies [8,48,49]. In our study, significantly weaker staining of 3β-HSD activity was detected in the high-dose (2.5 mg/kg) group, but both the 0.5 and 1.5 mg/kg CdCl_2_ treatments also exhibited inhibitory effects on 3β-HSD activity. In addition, previous studies have shown that 3β-HSD activity or expression is downregulated by Cd treatment [47,50]. Treatment with Cd also causes a significant decrease in serum testosterone levels [51]. A previous study indicated that testosterone levels are significantly impaired in animals exposed to 0.86 and 1.1 mg Cd/kg, although the density and morphology of Leydig cells are unchanged [52]. In our study, the activity of 3β-HSD is also significantly decreased by high dose of cadmium.

## 5. Conclusions

We found that the height of endothelial cells was significantly increased and blood vessel-associated proteins were upregulated with the administration of 2.5 mg CdCl_2_/kg/day compared with the control. It is possible that cadmium treatment may stimulate the proliferation of the mural cells and endothelial cells of blood vessels, which may lead to the testis functioning abnormally.

## Data Availability

Not applicable.
